# Transplant Trial Watch

**DOI:** 10.3389/ti.2024.13860

**Published:** 2024-10-14

**Authors:** Simon R. Knight, John M. O’Callaghan

**Affiliations:** ^1^ Centre for Evidence in Transplantation, Nuffield Department of Surgical Sciences, University of Oxford, Oxford, United Kingdom; ^2^ Oxford Transplant Centre, Churchill Hospital, Oxford, United Kingdom; ^3^ University Hospitals Coventry & Warwickshire, Coventry, United Kingdom

**Keywords:** randomised controlled trial, heart transplantation, everolimus, Bk virus, monoclonal antibody

To keep the transplantation community informed about recently published level 1 evidence in organ transplantation ESOT and the Centre for Evidence in Transplantation have developed the Transplant Trial Watch. The Transplant Trial Watch is a monthly overview of 10 new randomised controlled trials (RCTs) and systematic reviews. This page of Transplant International offers commentaries on methodological issues and clinical implications on two articles of particular interest from the CET Transplant Trial Watch monthly selection. For all high quality evidence in solid organ transplantation, visit the Transplant Library: www.transplantlibrary.com.

Randomised Controlled Trial 1 Long-Term Follow-Up of the Randomized, Prospective Scandinavian Heart Transplant Everolimus *De Novo* Study With Early Calcineurin Inhibitors Avoidance (SCHEDULE) Trial.

*by Bollano, E., et al. Journal of Heart and Lung Transplantation 2024 [record in progress]*.

## Aims

The aim of this study was to report the long term outcomes of calcineurin inhibitor (CNI) discontinuation and early initiation of everolimus in comparison to receiving a standard CNI-based regimen, in heart transplant recipients.

## Interventions

Participants were randomised to either the everolimus group or the CNI-group.

## Participants

115 adult *de novo* heart transplant recipients.

## Outcomes

The primary outcome was renal function. The secondary outcomes included time to death of any cause; a composite endpoint of death, myocardial infarction, re-transplantation, percutaneous coronary intervention (PCI), cancer, dialysis or kidney transplantation; myocardial structure and function; quality of life; and number of adverse events or serious adverse events.

## Follow-Up

12 years.

## CET Conclusion

*by John O’Callaghan*

This is an interesting study in heart transplantation with long follow up (11 years) after transplantation. It is the latest publication following the SCHEDULE trial in which heart transplant recipients were randomised to everolimus with reduced CNI exposure, followed by CNI withdrawal at week 7–11 post-transplant. This was compared to standard dose and continuation of CNI. Both these regimens were given alongside mycophenolate mofetil and corticosteroids. 78 of the 115 patients from the initial study were included at this follow up stage, with a very similar number from each arm of the study (40 versus 38). Analysis was by intention to treat, even at 11 years after randomisation, this is excellent allocation maintenance. Approximately 87% in each group were still on the allocated treatment. There was still a large statistically significant and clinically significant benefit for the everolimus group in terms of renal function (mean 83 mL/min versus 61 mL/min). There was no significant difference in heart transplant function, rejection or mortality. The earlier reports from this trial had raised concerns about increased risk of rejection in the everolimus group; however, this did not translate to later adverse outcomes in longer follow-up.

## Jadad Score

2.

## Data Analysis

Per protocol analysis.

## Allocation Concealment

Yes.

## Trial Registration

N/A.

## Funding Source

Industry and non-industry funded.

Randomised Controlled Trial 2 Nonclinical and Clinical Characterization of MAU868, A Novel Human-Derived Monoclonal Neutralizing Antibody Targeting BK Polyomavirus VP1.

*by Abend, J. R., et al. American Journal of Transplantation 2024 [record in progress]*.

## Aims

The aim of this study was to report *in vitro* and first in-human studies assessing the safety, tolerability and pharmacokinetics of MAU868, a novel human immunoglobulin (Ig) G1 monoclonal antibody, against BK polyomavirus VP1 in healthy adults.

## Interventions

Participants were randomised to receive either MAU868 or placebo.

## Participants

33 healthy adults (aged 18–60 years) weighing between 40 and 120 kg.

## Outcomes

The primary outcomes included safety and tolerability outcomes (adverse events and serious adverse events). Secondary outcomes were assessment of pharmacokinetics and the potential immunogenicity of MAU868.

## Follow-Up

182 days.

## CET Conclusion

*by Simon Knight*

This interesting paper reports *in vitro* and first-in-human studies of a novel human IgG1 monoclonal antibody against BK virus. *In vitro* studies demonstrate binding and neutralisation of infection with no evidence of resistance in long-term selection studies. The first-in-human clinical study in healthy volunteers demonstrated the pharmacokinetic profile of the drug, and the treatment was well tolerated with no major side effects at all doses. The clinical study was well designed, with randomisation and placebo control, although the exact method of randomisation and allocation concealment is not described. Given the lack of existing treatments for BK virus, this is a very promising initial study.

## Jadad Score

4.

## Data Analysis

Per protocol analysis.

## Allocation Concealment

No.

## Trial Registration

N/A.

## Funding Source

Industry funded.

## Clinical Impact Summary

*by Simon Knight*

BK virus infection, leading to viraemia and nephropathy, remains a significant issue in renal transplantation. Viraemia develops in up to 30% of kidney recipients, and despite improvements in care, infection still results in graft dysfunction and graft loss in around 15% affected patients [1, 2]. Most transplant centres monitor for presence of virus, either in the urine or blood, and respond to increasing levels of viraemia. A number of different approaches to management have been trialled, but mainstay of treatment remains immunosuppression reduction, sometimes with IvIG in patients who do not respond [3]. Antiviral therapy with leflunomide or cidofovir has not shown consistent benefit in randomised trials.

A lack of effective treatment for BK virus has led to two recent early-phase studies of novel therapies. Earlier this year, Chandraker and colleagues published a randomised, double blind safety study of Posoleucel, an allogeneic, multivirus specific T-cell therapy with activity against BK virus, adenovirus, cytomegalovirus, Epstein–Barr virus, human herpesvirus 6, and John Cunningham virus [4]. The treatment was shown to be safe and well-tolerated, and Posoleucel resulted in a reduction in BK viraemia compared to placebo with an increase in circulating BK-virus specific T-cells.

In the July issue of the American Journal of Transplantation, Abend and colleagues report a phase 1 study of a novel monoclonal antibody, MAU868, which targets the major capsid protein VP1 [5]. *In vitro* studies demonstrated binding and neutralisation of the 4 major BK virus genotypes, with no evidence of resistance in long-term selection studies. A first-in-human clinical study in healthy volunteers demonstrated the pharmacokinetic profile of the drug, and the treatment was well tolerated with no major side effects at all doses tested.

Clinical studies of both of these drugs are in early stages, and further clinical trials will be needed to demonstrate whether the activity against BV viraemia results in sustained viral reduction and improved clinical outcomes. Nonetheless, it is encouraging to see the development of new agents against this problematic virus.

### Clinical Impact

3/5.
